# Restoring the Balance: CRISPRa-Driven β-Tubulin Compensation as a Strategy for Tubulinopathy Treatment

**DOI:** 10.3390/ijms27156954

**Published:** 2026-08-03

**Authors:** Sydney Steiman, Ciara Kinsella, Yixiang Zhang, Safia Omer, Cadence Brown, Georgiana Forguson, Lujaina Elbakr, Stephen Pastore, Evgueni A. Ivakine

**Affiliations:** 1 Genetics and Genome Biology Program, The Hospital for Sick Children, Toronto, ON M5G 0A4, Canada; 2 Department of Physiology, University of Toronto, Toronto, ON M5S 1A8, Canada; 3 Department of Molecular and Cellular Biology, University of Guelph, Guelph, ON N1G 2W1, Canada; 4 Department of Molecular Genetics, University of Toronto, Toronto, ON M5S 1A8, Canada

**Keywords:** tubulinopathy, ciliopathy, CRISPR activation

## Abstract

Microtubules are essential cytoskeletal components comprising alpha- and beta-tubulin proteins that facilitate organelle positioning, cell migration, division, and intracellular trafficking. Mutations in tubulin genes can lead to tubulinopathies, a class of rare genetic neurodevelopmental disorders characterized by a range of brain malformations and other clinical features. Recent studies have shown that pathogenic variants in beta-tubulin genes such as TUBB^G308S^ have been found to underlie the development of ciliopathies, disorders impacting cilia, important organelles for development and cell motility. Thus, mutations in distinct tubulin genes, which present a significant hurdle for the development of therapeutic gene editing strategies and targeted therapeutics. Here, we describe the development of a mutation-independent treatment strategy based on the upregulation of non-mutated beta-tubulin isotype protein using CRISPR-Cas9 activation. By increasing the expression of various beta-tubulin proteins, we demonstrate a restoration of the microtubule network and primary cilia formation.

## 1. Introduction

The human genome contains many gene families that encode functionally redundant proteins [1]. Examples include the tubulin family, laminin alpha family, Hox gene clusters, Fibroblast growth factor receptors, Kinesin family, Dynein chains, and Actin family [2,3,4,5,6]. Modelling and treating mutations in highly homologous, functionally redundant gene families has proven particularly challenging [7]. Although recent advances in gene therapy have enabled effective treatment for monogenic disorders such as Sickle Cell Disease, applying these approaches to diseases involving large gene families remains a hurdle [8,9]. This challenge underscores the need for alternative and innovative therapeutic strategies to address disorders caused by dysfunction within large gene families.

**Figure 1:**
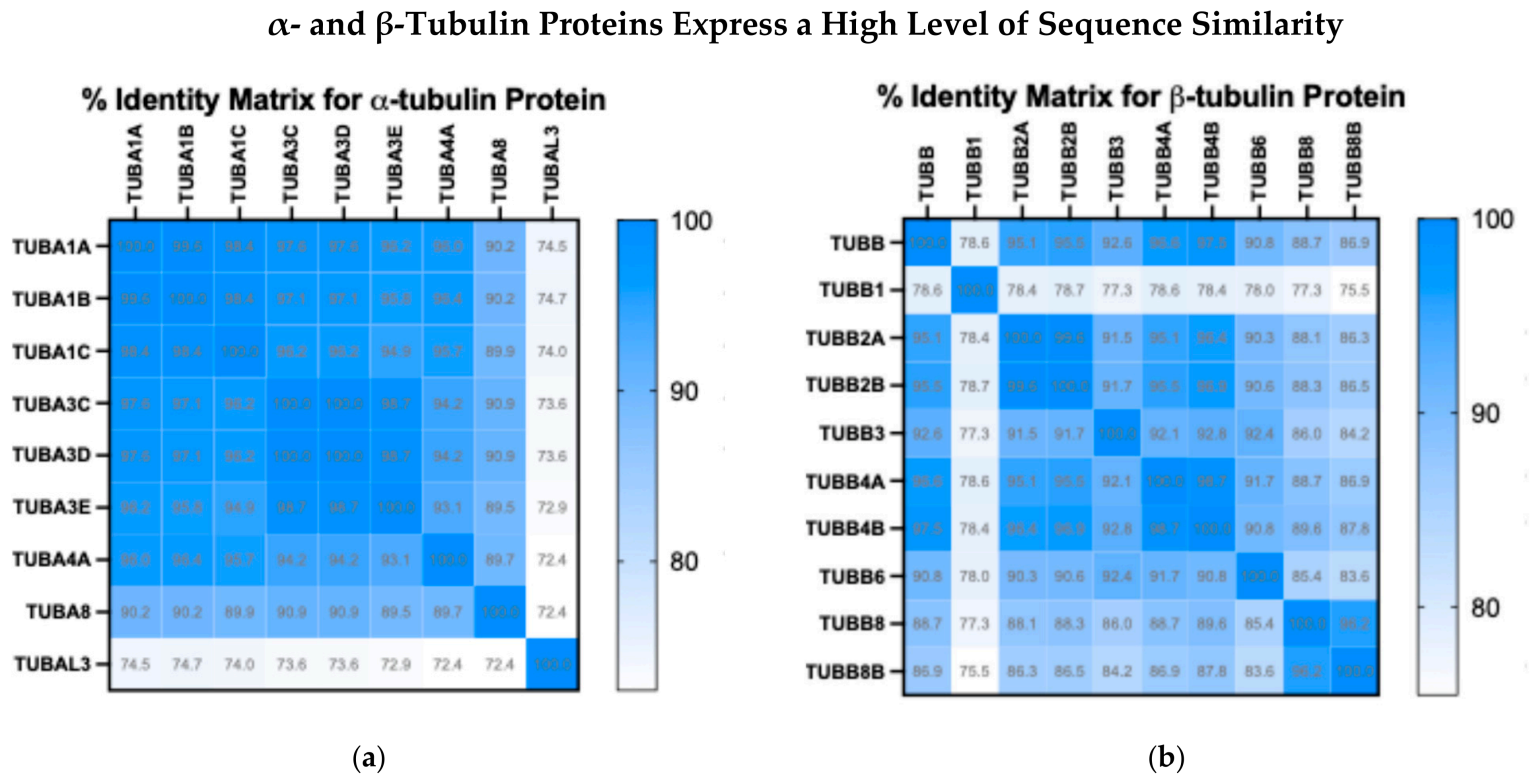
A percent identity matrix of (**a**) α-tubulin and (**b**) β-tubulin amino acid sequence reveals a high degree of sequence similarity, highlighting the challenges in genome editing strategies for modelling specific tubulinopathy variants [18].

The tubulin superfamily consists of diverse globular proteins vital for a wide range of cell processes [10]. In eukaryotes, six distinct tubulin families have been identified, each comprising multiple isotypes that are hypothesized to originate from duplication events throughout evolution. The expression patterns of these families vary across species, with some species having additional family members and others lacking certain tubulin types [11,12]. For example, zeta-tubulin is associated with the basal foot, a centriolar appendage that connect centrioles to the apical cytoskeleton and is absent in humans but present in *Xenopus* [13]. In humans, tubulin is composed of nine isotypes for α-tubulin and 10 isotypes for β-tubulin, among other tubulins [14]. These α- and β-tubulin subunits heterodimerize to assemble the extensive filamentous intracellular microtubule network [15]. Amongst the isotypes of α- and β-isotypes, there is a large degree of sequence similarity and functional redundancy, creating significant challenges for genetic modelling and genome editing approaches (Figure 1) [16]. The expression of α- and β-isotypes throughout brain development, in adult brains, and across tissue types is unique. For example, TUBB3 is constitutively expressed in neurons, TUBB is predominately expressed during early brain development, and TUBB1 expression is limited to hematopoietic tissues [17].

Tubulinopathies comprise a class of rare genetic neurodevelopmental disorders caused predominantly by de novo heterozygous missense variants in tubulin genes [19,20]. To date, seven tubulin genes have been implicated in cerebellar malformations such as agenesis and cerebellar hypoplasia or dysplasia: *TUBA1A*, *TUBA8*, *TUBB2A*, *TUBB2B*, *TUBB3*, *TUBB*, and the γ-tubulin isotype *TUBG1* [21,22]. A previous study by our lab identified a de novo heterozygous missense variant (p.G308S) in Tubulin Beta Class I (*TUBB*) with features of Joubert syndrome, a primary ciliopathy, and tubulinopathy. Currently, no effective therapies are available for patients with tubulinopathy diseases [19].

In addition to its applications in precise genome editing, the CRISPR/Cas9 system has been extensively engineered as a platform for transcriptional and epigenetic modulation [23,24]. Introducing mutations into the nuclease domains of Cas9 abolished its DNA cleavage activity while preserving sg-RNA-guided DNA binding capabilities [25,26]. This catalytically inactive form of Cas9, termed dead Cas9 (dCas9), was initially developed for CRISPR interference (CRISPRi)-mediated gene repression. Subsequent engineering efforts led to the fusion of transcriptional activation domains to dCas9, enabling the development of CRISPR activation (CRISPRa) systems for targeted endogenous gene upregulation [27,28]. Several CRISPRa architectures have since been optimized, including the VPR system, which incorporates the VP64, p65, and Rta activation domains fused in tandem to the C-terminus of dCas9. A second-generation platform, the synergistic activation mediator (SAM), utilizes dCas9-VP64 in combination with an engineered scaffold RNA (scRNA) containing MS2 RNA aptamers. This system recruits MS2 bacteriophage coat proteins fused to the transcriptional activators p65 and heat shock factor 1 (HSF1) [29,30].

During the development and optimization of CRISPRa, it was observed that the simultaneous delivery of multiple sgRNAs targeting distinct regions within a gene’s promoter or transcriptional regulatory elements, such as an enhancer, substantially enhanced gene activation compared with single-guide approaches [31,32]. This is caused by the synergistic recruitment of transcriptional machinery to promoter/regulatory regions. Building upon this system, several groups have generated optimized genome-wide CRISPRa sgRNA libraries targeting thousands of genes, thus enabling scalable and high-throughput activation screens [30,33]. Compared with other CRISPR-Cas9-based technologies, CRISPRa offers a unique approach to genetic therapy that is independent of underlying mutations and unaffected by target gene size. Moreover, with the catalytically inactive nature of dCas9, the risk of sequence insertions, deletions and off-target effects remains low [34,35].

High sequence similarity among members of large gene families complicates precise editing because homologous genes can share sgRNA binding sites, resulting in off-target cleavage. CRISPRa has been utilized to increase the expression of structurally similar proteins such as *LAMA1* to rescue muscular dystrophy phenotypes caused by mutations or deficiencies in the *LAMA2* gene [36,37]. This example highlights how CRISPRa is a powerful tool with the potential to treat mutationally heterogeneous patient populations by upregulating the expression of a single homologous gene. Here we present a strategy for the CRISPRa-based modulation of β-tubulin genes using an established tubulinopathy model.

Using the TUBB^G308S^ cell model, we investigated whether optimized sgRNAs in combination with CRISPRa could rescue disease-associated phenotypes previously characterized in this variant. The goal was to establish a framework for developing gene therapies for tubulinopathies by leveraging the functional redundancy that exists among members of the tubulin gene family.

## 2. Results

### 2.1. CRISPR Activation Domains Schematic and Downstream sgRNA Targets

**Figure 2:**
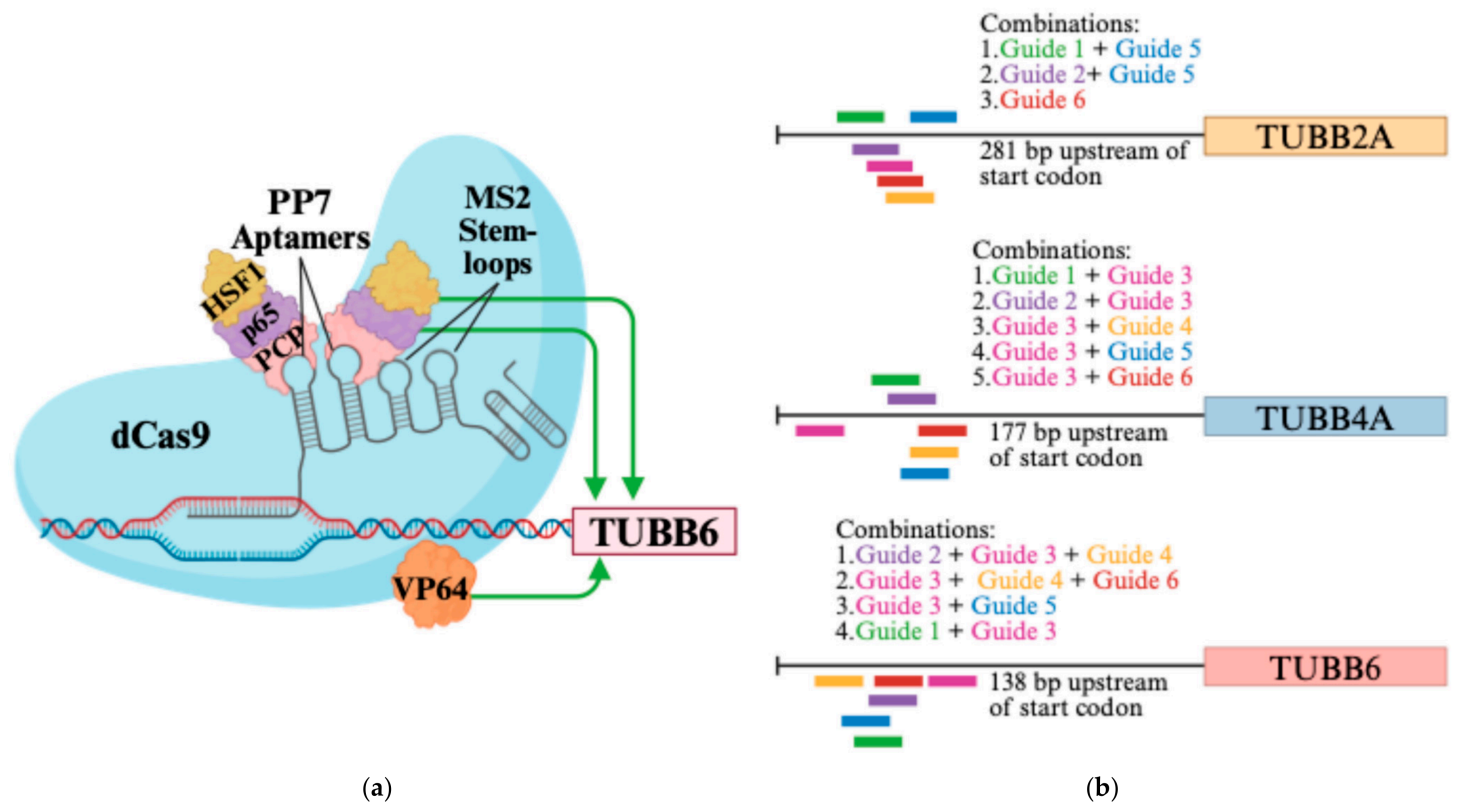
CRISPR activation machinery. (**a**) Schematic of the CRISPR activation complex when bound to target DNA; (**b**) schematic of sgRNA upstream of TUBB2A, TUBB4A, and TUBB6. Created in BioRender. Forguson, G. (2026) https://BioRender.com/9f0to4d (accessed on 29 July 2026).

To establish a system for CRISPRa-based compensation of β-tubulin genes as a therapeutic strategy, we engineered a sgRNA system targeting proximal promoter regulatory regions of alternative β-tubulin family members. For subsequent experiments, we employed a well-established and optimized CRISPRa system together with a sgRNA pool (Figure 2a) [30]. This approach was designed to maximize the transcriptional induction of target genes while preserving genomic integrity through non-cutting modulation of DNA.

We investigated different β-tubulin isotypes expressed in our TUBB^G308S^ cell model, *TUBB2A*, *TUBB4A*, and *TUBB6*. Six candidate sgRNAs were selected from the Calabrese CRISPRa library for each target gene. These sgRNAs were designed to target defined regulatory regions upstream of the transcriptional start site. To identify the optimal activation strategy for each isotype, multiple sgRNA combinations were selected based on target site occupancy, with the aim of minimizing competitive binding and steric hinderance between simultaneously recruited dCas9 transcriptional activator complexes (Figure 2b).

### 2.2. Assessing CRISPRa sgRNA Transcriptional Upregulation Efficiency in the TUBB^G308S^ Cell Line

**Figure 3:**
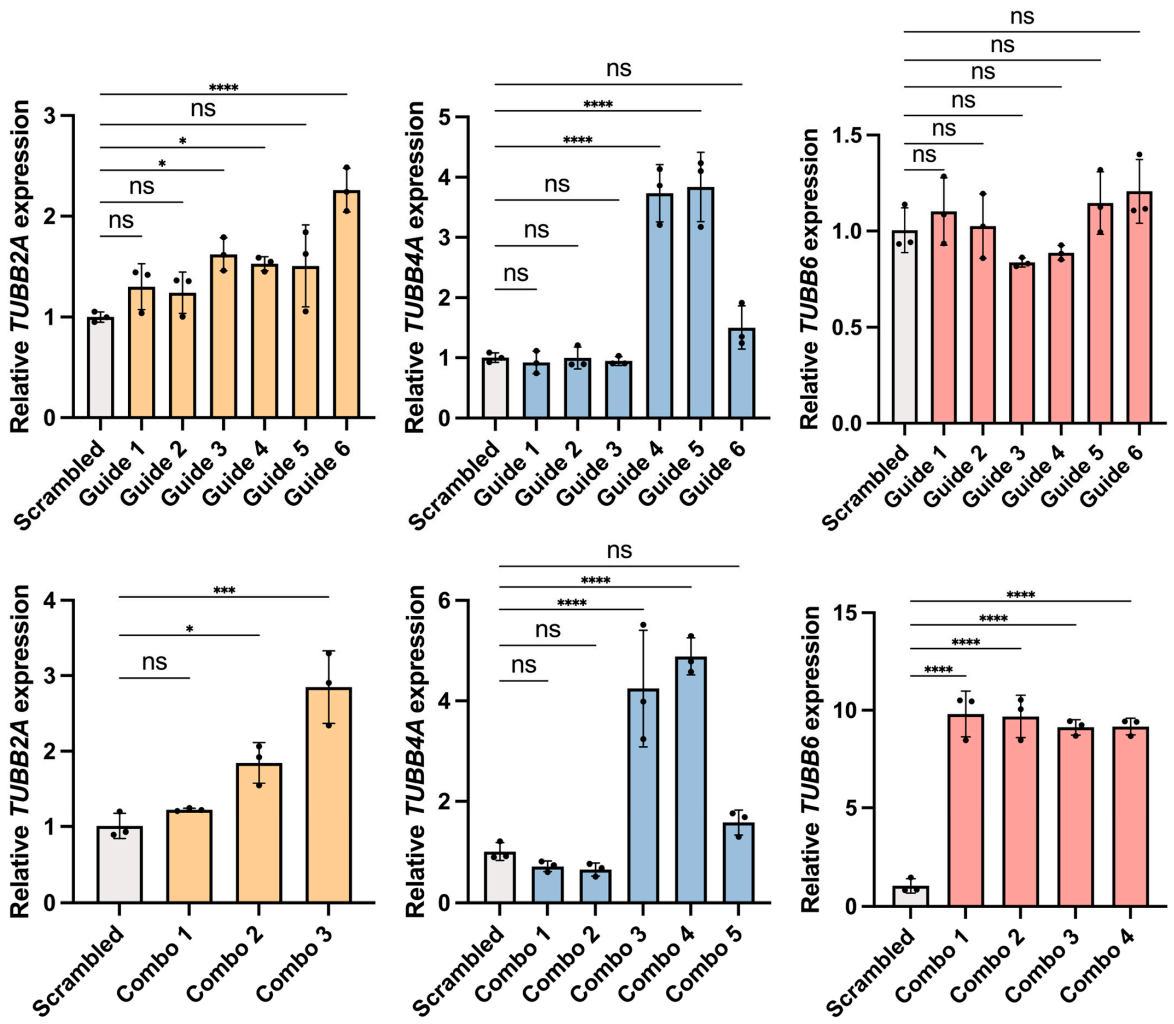
qRT-PCR of *TUBB2A*, *TUBB4A*, and *TUBB6* single sgRNA and combinatorial sgRNA levels in the TUBB^G308S^ cell line. (*n* = 3 lysates per condition; data are presented as mean ± SD. Statistical significance was determined using ordinary one-way ANOVA. Homogeneity of variances was assessed using the Brown–Forsythe test and Bartlett’s test. Ns, not significant (*p* > 0.05), * *p* < 0.05, *** *p* < 0.001, **** *p* < 0.0001). Created in BioRender. Forguson, G. (2026) https://BioRender.com/9f0to4d (accessed on 29 July 2026).

To evaluate the efficacy of CRISPRa-mediated activation of *TUBB2A*, *TUBB4A*, and *TUBB6*, we performed qRT-PCR to determine whether the delivery of individual sgRNAs or multiplexed sgRNA combinations resulted in increased expression of target β-tubulin transcripts. Relative to a scrambled sgRNA control, only a subset of individual sgRNAs induced significant upregulation of their respective target genes. Specifically, *TUBB2A* sgRNA 3, 4, and 6, as well as *TUBB4A* sgRNA 4 and 5, produced a significant increase in β-tubulin transcript expression (Figure 3).

Given the limited activation observed with the single-guide construct strategy, we next investigated whether transcriptional induction could be enhanced using a multiplexed sgRNA delivery. This approach was designed to increase the recruitment of the dCas9 transcriptional activator complex and enhance the accumulation of local transcriptional machinery at the transcriptional start site. qRT-PCR analysis identified distinct high-performing sgRNA combinations for *TUBB2A*, *TUBB4A*, and *TUBB6*. These combinations were subsequently selected for downstream functional investigation (Figure 3). Interestingly, *TUBB6* sgRNAs (combinations 1–4) produced the most increase in fold change compared to the corresponding combinations targeting the other β-tubulin isotypes. This finding suggests that *TUBB6* may be particularly amenable to CRISPRa-mediated transcriptional activation in this cellular context.

### 2.3. Investigating Microtubule Intensity in the CRISPRa Treated TUBB^G308S^ Cell Line

**Figure 4:**
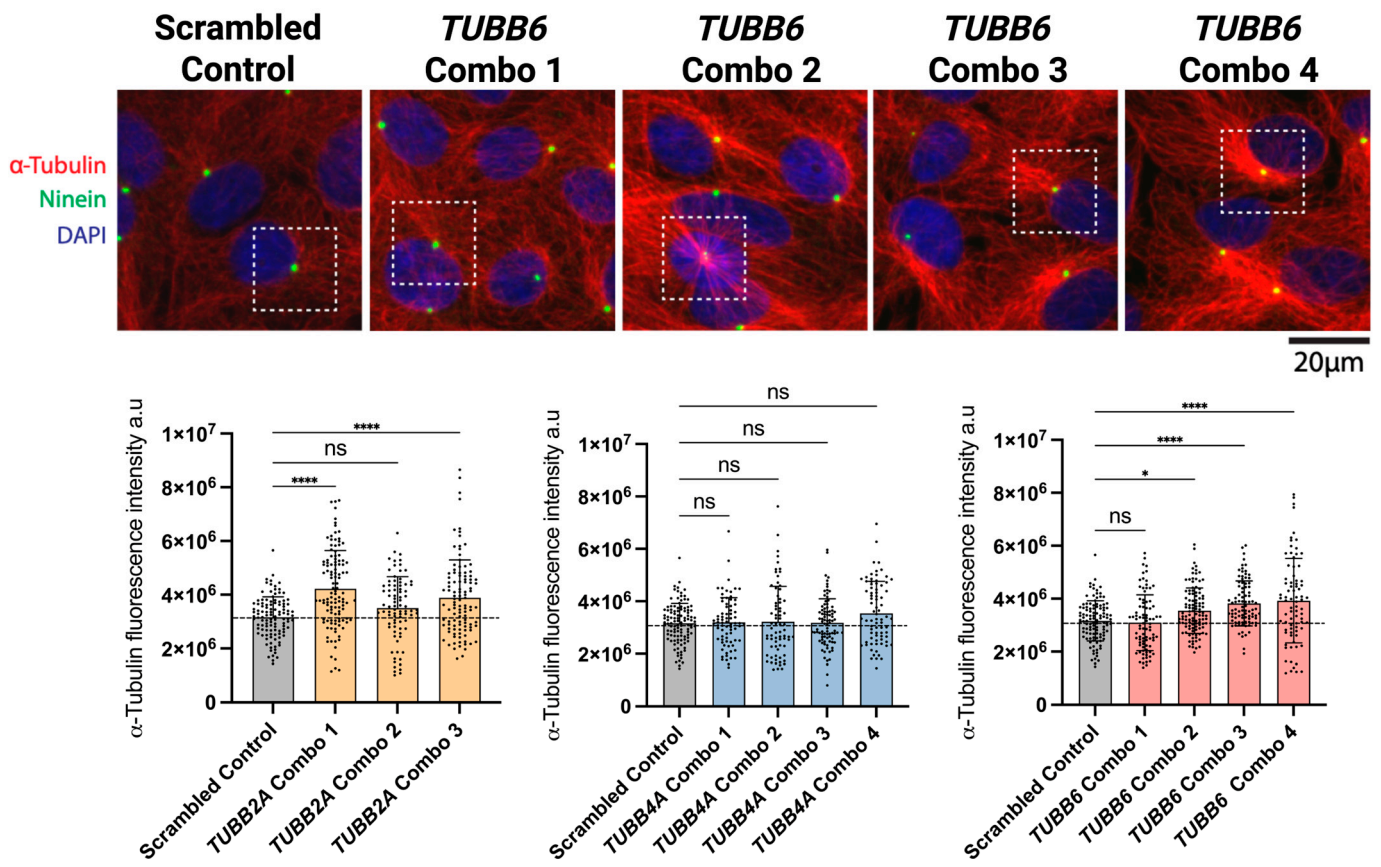
Microtubule intensity changes with CRISPRa combinatorial guide treatment in the TUBB^G308S^ cell line. Representative images of the α-tubulin network are shown in red, with the ninein centrosomal marker in green. Scatter plot of fluorescence intensity of ninein; data are from three experiments and represented as mean ± SD. Statistical significance was determined using ordinary one-way ANOVA. Homogeneity of variances was assessed using the Brown–Forsythe test and Bartlett’s test. Ns, not significant (*p* > 0.05), (* *p* < 0.05, *** *p* < 0.001, **** *p* < 0.0001). Created in BioRender. Forguson, G. (2026) https://BioRender.com/9f0to4d (accessed on 29 July 2026).

Previous characterization of the TUBB^G308S^ cell line demonstrated reduced α-tubulin fluorescence intensity surrounding the ninein-marked centrosome, suggesting impaired microtubule nucleation and altered polymerization dynamics [19]. To determine whether CRISPRa-mediated β-tubulin compensation could restore microtubule organization, optimized sgRNA combinations targeting *TUBB2A*, *TUBB4A*, and *TUBB6* were applied. Subsequent α-tubulin fluorescence intensity was assessed by confocal microscopy and immunofluorescence analysis to determine whether CRISPRa treatment could rescue the dysregulated microtubule network previously identified in this model (Figure 4).

Following CRISPRa treatment, several sgRNA combinations resulted in increased α-tubulin fluorescence intensity surrounding the centrosomal region relative to scrambled sgRNA controls. This increase suggests a partial restoration of microtubule nucleation and organization. Notably, the extent of rescue varied among the targeted β-tubulin isotypes, indicating potential differences in their capacity to functionally compensate for the pathogenic TUBB^G308S^ variant. These findings further support the functional compensatory potential of alternative β-tubulin isotypes in rescuing cytoskeletal defects associated with pathogenic variants in TUBB. These results further highlight the therapeutic potential of CRISPR-based transcriptional activation as a strategy for addressing disorders caused by dysfunction within highly redundant gene families.

### 2.4. Assessing Microtubule Curvature in the TUBB^G308S^ Cell Line with CRISPRa

**Figure 5:**
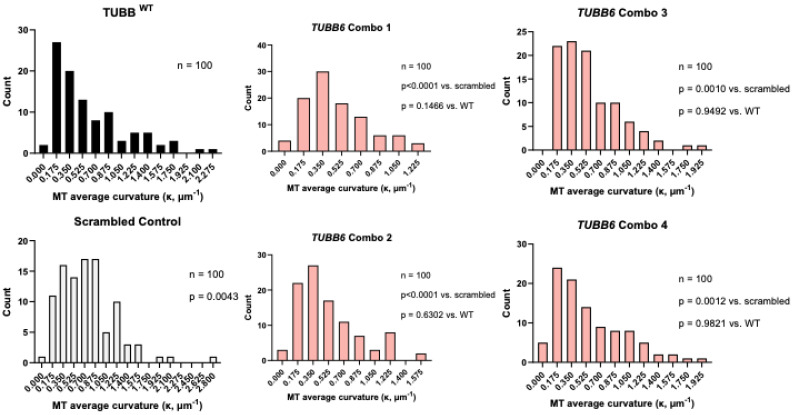
Microtubule curvature improves with *TUBB6* CRISPRa combinatorial guide treatment in the TUBB^G308S^ cell line. Statistical significance was determined using ordinary one-way ANOVA with Tukey’s multiple comparisons test for WT and scrambled comparisons. The Mann-Whitney test was used to calculate *p* values for comparing *TUBB6* CRISPRa combinations and scrambled control compared to WT.

Since *TUBB6* combos 1–4 showed improvements in microtubule intensity, we next sought to investigate microtubule curvature. The TUBB^G308S^ cell model displays excessive microtubule curvature in comparison to a wild-type control. The distribution of the average microtubule curvature was shifted to the right. This suggests that the variant tubulin protein can negatively alter the microtubule lattice and decrease filament organization. The rescue of microtubule curvature observed with all four *TUBB6* CRISPRa combinations highlights another phenotypic rescue of this therapeutic platform and suggests that the microtubule lattice is being partially restored with wild-type TUBB6 expression (Figure 5).

### 2.5. Investigating Primary Cilia Restoration in the CRISPRa Treated TUBB^G308S^ Cell Line

We next investigated whether CRISPRa-mediated β-tubulin compensation could rescue defects in primary ciliogenesis in the TUBB^G308S^ cell model. Optimized sgRNA combinations targeting *TUBB2A*, *TUBB4A*, and *TUBB6* were delivered to the TUBB^G308S^ variant cell line, which exhibits reduced primary cilia formation [19]. Following delivery of dCas9 and sgRNA constructs, the proportion of ciliated cells was quantified and compared to TUBB^G308S^ cells treated with a scrambled sgRNA control.

**Figure 6:**
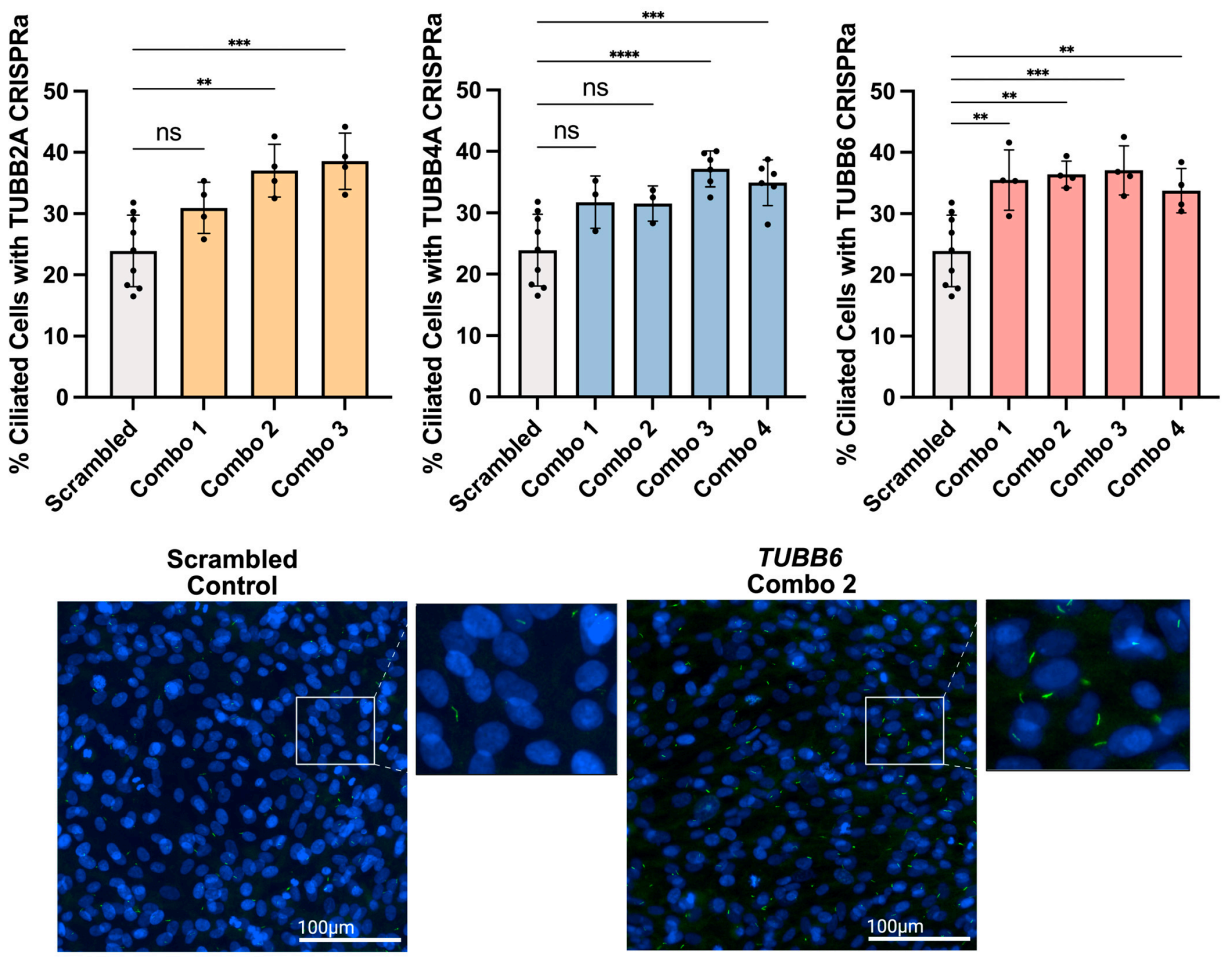
Quantification or primary cilia with CRISPRa treatment in the TUBB^G308S^ cell model. Representative images below for scrambled control and *TUBB6* combo 2 with nuclei in blue (DAPI) and cilia in green (ARL13B). (Data are presented as mean ± SD. Statistical significance was determined using ordinary one-way ANOVA. Homogeneity of variances was assessed using the Brown–Forsythe test and Bartlett’s test. Ns, not significant (*p* > 0.05), ** *p* < 0.01, *** *p* < 0.001, **** *p* < 0.0001). Created in BioRender. Forguson, G. (2026) https://BioRender.com/9f0to4d (accessed on 29 July 2026).

CRISPRa treatment resulted in a significant increase in primary cilia formation for several sgRNA combinations targeting distinct β-tubulin isotypes (Figure 6). Notably, sgRNA combinations that failed to increase β-tubulin transcript abundance (Figure 3) also failed to rescue ciliogenesis. In contrast, guide combinations that produced a more robust transcriptional activation were associated with enhanced primary cilia formation. These included *TUBB2A* sgRNA combinations 2 and 3, *TUBB3* sgRNA combination 3 and 4, and *TUBB6* sgRNA combinations 1, 2, 3, and 4. Collectively, these findings suggest that compensatory upregulation of alternative β-tubulin isotypes can functionally restore certain aspects of primary ciliogenesis in tubulinopathy models. Importantly, the correlation between increased β-tubulin expression and improved ciliogenesis suggests that CRISPRa-mediated upregulation of alternative β-tubulin isotypes can functionally compensate for the pathogenic effects of the TUBB^G308S^ variant. Collectively, these findings demonstrate that transcriptional activation of homologous β-tubulin genes can restore key cellular phenotypes in a tubulinopathy model, further supporting the potential of CRISPRa-based compensation as a therapeutic strategy for disorders arising from mutations within highly redundant gene families.

### 2.6. CRISPRa-Treated TUBB^G308S^ Cell Show Normal Primary Cilia Length

**Figure 7:**
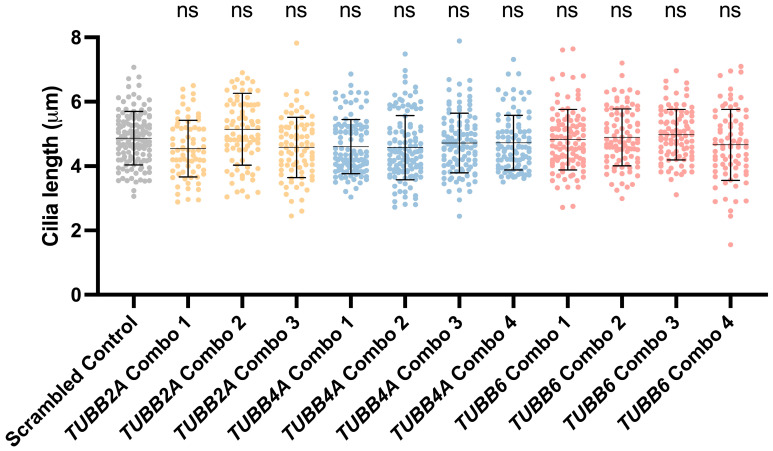
Primary cilia length with CRISPRa treatment in the TUBB^G308S^ cell model. (For each sample *n* ≥ 60, data are presented as mean ± SD. Statistical significance was determined using ordinary one-way ANOVA. Homogeneity of variances was assessed using the Brown–Forsythe test and Bartlett’s test. Ns, not significant (*p* > 0.05)).

Next, we sought to determine whether CRISPRa-mediated β-tubulin compensation influenced ciliary morphology. Primary cilia length was quantified following treatment with optimized sgRNA combinations targeting alternative β-tubulin isotypes. Immunostaining analysis revealed no overt changes in primary cilia length across any treated conditions when compared to scrambled sgRNA controls (Figure 7). These findings indicate that while CRISPRa-mediated upregulation of alternative β-tubulin genes promotes ciliogenesis and increases the proportion of ciliated cells, it does not substantially alter ciliary length once the cilia are formed. Importantly, the absence of aberrant ciliary elongation of shortening suggests that CRISPRa-based β-tubulin compensation restores cilia formation without disrupting normal ciliary morphology. Collectively, these results demonstrate that transcriptional activation of alternative β-tubulin isotypes can rescue defects in primary cilia formation in the TUBB^G308S^ cell model while maintaining ciliary architecture. This further supports the therapeutic potential of CRISPRa-mediated gene compensation as a strategy for correcting cellular phenotypes associated with tubulinopathies.

## 3. Discussion

Despite the compensatory and functionally redundant nature of α- and β-tubulin isotypes, CRISPRa-based therapeutic strategies have not yet been explored in the context of tubulinopathies. Here, we demonstrate that targeted transcriptional activation of β-tubulin isotypes can partially rescue key cellular phenotypes in a tubulinopathy and ciliopathy model TUBB^G308S^.

Using a combinatorial sgRNA strategy, we achieved robust upregulation of selected β-tubulin isotypes, including *TUBB2A*, *TUBB4A*, and *TUBB6*. These isotypes display high amino acid sequence similarity (Figure 1) to *TUBB* and were chosen due to their tissue specific and intracellular roles. *TUBB2A* and *TUBB4A* are isotypes highly expressed in the brain and central nervous system and are associated with neurodegenerative diseases [38,39,40,41]. TUBB6 is also an isotype documented to have critical roles in microtubule organization and dynamics [42,43]. The combinatorial sgRNA approach consistently outperformed single-guide activation strategies, suggesting that coordinated start site engagement enhances transcriptional output for specific β-tubulin loci. This approach is also shown to enhance activation in mammalian cells for genes such as *SOX2* and *IL1RN* [44]. Functionally, CRISPRa-mediated isotype upregulation was associated with partial correction of cytoskeletal abnormalities in Figure 4 and restoration of primary cilia formation exhibited in Figure 5. In addition, the primary cilia formed in sgRNA-treated TUBB^G308S^ cells exhibited normal length, indicating that recovery appears to preserve ciliary structural integrity.

To our knowledge, this represents the first application of CRISPRa-mediated transcriptional modulation as a therapeutic strategy for tubulinopathies arising from heterozygous missense variants in TUBB. Beyond its therapeutic implications, this platform provides a framework for dissecting functional redundancy and specialization among β-tubulin isotypes in a tubulinopathy disease-relevant context.

The TUBB^G308S^ cell model additionally recapitulates phenotypic features of the primary ciliopathy Joubert syndrome, supporting emerging evidence that tubulinopathies and ciliopathies exist along a shared pathobiology spectrum [14,19]. Increasing recognition of overlapping clinical and mechanistic features suggests that alterations in microtubule composition and function can converge on ciliary dysfunction and downstream neurodevelopmental pathology. Within this context, variant-agnostic transcriptional activation represents a potentially generalizable strategy for tubulin-associated diseases. Accordingly, additional TUBB variants associated with ciliary dysfunction warrant systematic evaluation using this approach.

The rescue of ciliogenesis, microtubule intensity, and microtubule curvature in the TUBB^G308S^ cell model following CRISPRa-mediated β-tubulin isotype upregulation may reflect a reversal of potential pathogenic mechanisms that arise in missense TUBB variants. The increase in wild-type β-tubulin transcripts detected by qRT-PCR may facilitate the correct α/β-tubulin heterodimerization into the microtubule lattice, which could partially compensate for structural defects introduced by the G308S variant. Additionally, rebalancing α/β-tubulin dimer availability and the altered isotype composition may influence microtubule dynamics, including rates of catastrophe and polymer stability. Reduced microtubule instability could indirectly support ciliary axoneme assembly and maintenance. The proper regulation of primary cilia length is essential for maintaining proper signal transduction and signalling pathways. Shortened cilia may limit pathway activities, while elongated primary cilia could disrupt ciliary trafficking and structural stability. Consequently, both shortened and elongated primary cilia length can contribute to ciliopathy-associated phenotypes [45,46].

While our transient delivery of CRISPRa machinery for transcriptional activation offers a temporary and variant unspecific approach, global upregulation of β-tubulin isotypes may have broader consequences on cytoskeletal homeostasis. Excess tubulin availability may alter microtubule polymerization dynamics, spindle function, and intracellular trafficking [47]. This underscores the need for future considerations such as dose optimization and cell-type specificity. The translation of CRISPRa-based therapeutics for the treatment of tubulinopathies would need to address delivery constraints associated with large dCas9 systems, specifically in the context of central nervous system delivery [48,49,50].

Due to the size of our current CRISPRa strategy with dCas9 and combinatorial guides, delivery using an AAV-based method would require a dual AAV system splitting up the dCas9 and the sgRNAs to overcome the packaging limits [51]. Additionally, AAV-based CRISPR-Cas9 genome editing faces many potential challenges for translation including patient immune responses, tissue selectivity, and off-target editing [52]. While CRISPRa would not induce off-targeting edits, these virus-based delivery systems would require extensive optimization. Alternative delivery methods such as lipid nanoparticles have shown promise for efficient CRISPR-Cas9 delivery in immune system evasion and prolonged circulation and should be considered as potential vehicles for delivery [53].

In addition to the developmental sensitivity of cilia-facilitated signalling pathways such as Shh, future work should prioritize tissue-specific regulation and compact delivery system optimization. Furthermore, while primary cilia are found across different tissues ranging from the brain to kidneys, the potential rescue capacity of CRISPRa could vary amongst tissue types where β-tubulin isotype patterns can differ [54,55]. Specifically, *TUBB1* is hematopoietic cell-specific, whereas isotypes such as *TUBB2A*, *TUBB2B*, *TUBB3*, and *TUBB4A* are highly expressed in the brain [56,57].

Collectively, the findings in this study establish a transcriptional reprogramming approach to β-tubulin isotypes as a potential therapeutic strategy for tubulinopathies and ciliopathies with underlying variants in TUBB. Rather than correcting individual variants, this approach suggests a broader paradigm in which functional redundancy among β-tubulin isotypes can be harnessed to restore cellular architecture and overcome disease phenotypes.

## 4. Materials and Methods

### 4.1. CRISPR Activation sgRNA Design and Generation

SpCas9 sgRNAs were cloned into pXPR_502 (Addgene #96923). sgRNAs were chosen from the Human CRISPR Activation Pooled Library (Calabrese VP64) with gRNA 1–3 from Set A (Addgene Cat#92377) and gRNA 4–6 from Set B (Addgene Cat#92378). Lenti dCAS-VP64_Blast was a gift from Feng Zhang (Addgene plasmid # 61425; http://n2t.net/addgene:61425 (accessed on 1 April 2025); RRID:Addgene_61425).

### 4.2. General Plasmid Preparation

Plasmid-based delivery of CRISPR constructs to hTERT-RPE1 cells was used. Competent Stbl3 *Escherichia coli* cells (prepared in-house by calcium chloride) were transformed with plasmids by heat shock, after which bacterial cells were plated and grown overnight at 37 °C on Luria broth (LB) agar plates with carbenicillin (50 μg/mL) per plasmid-conferred resistance. Subsequent colonies were inoculated in LB with carbenicillin and further cultured for 12–15 h. Plasmid DNA was isolated using the NucleoBond Xtra Midi kit from Macherey-Nagel(Macherey-Nagel, Düren, Germany).

### 4.3. General Cell Culture Conditions

p53-null hTERT-RPE1 cells (a gift from Daniel Durocher) were cultured in Dulbecco’s Modified Eagle Medium (DMEM)/Nutrient Mixture F-12 1:1 Mix (Thermo Fisher Scientific 10565018 (Thermo Fisher Scientific, Waltham, MA, USA)) supplemented with 10% heat-inactivated FBS (Wisent Bioproducts 080-450 (Wisent Inc., Saint-Jean Baptiste, QC, Canada)) and 1% penicillin–streptomycin solution (Wisent Bioproducts 450-201-EL) and then maintained at 37 °C in a humidified atmosphere (5% CO_2_). Where indicated, serum starvation was performed by washing cells twice with pre-warmed PBS (Wisent Bioproducts 311-010-CL) and then culturing them in serum-free Opti-MEM^TM^ (Thermo Fisher Scientific 31985-062) for 48 h.

Cells were electroporated using the Neon^TM^ Transfection System (Thermo Fisher Scientific MPK5000) with a pulse voltage of 1050 V, a pulse width of 30 ms, and a pulse number of 2. Cells were electroporated with 200,000 cells/well on 12-well plates (Corning 3513 (Corning Inc., Corning, NY, USA)), and media was replaced 24 h after plating. Electroporation efficiency is routinely tested with an EGFP construct to validate a 75% efficiency.

The TUBB^G308S^ heterozygous hTERT-RPE1 cell lines were generated by prime editing 3b (PE3b) and have been extensively described previously. In brief, the TUBB^G308S^ hTERT-RPE1 cell line was generated from p53-null hTERT-RPE1 cells (a gift from Daniel Durocher) to achieve isogenic cell lines detectable by Sanger sequencing. Cells were transfected with Lipofectamine™ 3000 Transfection Reagent (Thermo Fisher Scientific L3000008) following the manufacturer’s recommended protocol, with 845 ng of pegRNA-expression plasmid, 280 ng of the nicking gRNA and blasticidin resistance-expression plasmid, and 375 ng of the prime editor-expression plasmid (pCMV-PE2). Next, 24 h post-transfection blasticidin (Thermo Fisher Scientific A1113903) was added at 30 μg/mL in growth media for 6 days, with the media replaced daily. Cells were single-cell sorted using a MoFlo XDP cell sorter (Beckman Colter (Beckman Colter, Brea, CA, USA)), and clonal lines were expanded; genomic DNA was extracted to determine editing success.

### 4.4. Antibodies

For immunostaining experiments, the following primary antibodies were used: mouse monoclonal anti-α-tubulin (DM1A, Sigma-Aldrich T6199, 1:500 dilution (Sigma-Aldrich, St. Louis, MO, USA)), rabbit polyclonal anti-ninein (Sigma-Aldrich ABN1720, 1:300 dilution), and rabbit polyclonal anti-ARL13B (Proteintech 17711-1-AP, 1:300 dilution (Proteintech, Rosemont, IL, USA)).

The following secondary antibodies were used: goat anti-rabbit IgG (H + L) Alexa Fluor™ 488 (ThermoFisher A-11008), goat anti-rabbit IgG (H + L) Alexa Fluor™ 555 (ThermoFisher A-21429), goat anti-mouse IgG (H + L) Alexa Fluor™ 488 (ThermoFisher A-11001), goat anti-mouse IgG (H + L) Alexa Fluor™ 555 (ThermoFisher A-21422).

### 4.5. RNA Isolation and Quantitative RT-PCR

Total RNA isolation was performed using the RNeasy Mini Kit (Qiagen 74106 (Qiagen, Venlo, The Netherlands) according to the manufacturer’s recommended protocol with 1 μg of total RNA being used to reverse transcribe to cDNA. Reverse transcription was performed using SuperScript™ III Reverse Transcriptase (Thermo Fisher Scientific 18080044) following the manufacturer’s recommended protocol, with a 20 µL reaction volume.

Quantitative RT-PCR (qRT-PCR) was performed on a 1:10 dilution of the template cDNA using PowerUp™ SYBR™ Green Master Mix (Thermo Fisher Scientific A25776) in a QuantStudio™3 Real-Time PCR System (Thermo Fisher Scientific, Applied Biosystems A28136 (Applied Biosystems, Foster City, CA, USA)). The relative expression levels of each beta-tubulin gene was compared using the ∆∆Ct method, with *GAPDH* used as a housekeeping control for expression normalization.

### 4.6. Immunofluorescence

Cultured cells were plated on glass coverslips (VWR^®^ Coverslips 18 mm Circle No.1 48380-046) in 12-well tissue culture-treated plates (Corning 3513). Cells were washed once with PBS (Wisent Bioproducts 311-010-CL) and then fixed in ice-cold methanol (Fisher Scientific A412-4) at −20 °C for 15 min. Cells were then washed twice with PBS and blocked with 5% FBS in PBS. Coverslips were incubated with the primary antibodies listed above diluted in a blocking buffer at 4 °C overnight in a humidified chamber. Coverslips were then washed three times with PBS and incubated at room temperature for 1 h with the appropriate Alexa Fluor™-conjugated secondary antibody (1:500 dilution in a blocking buffer). Cover slips were then counterstained with Hoechst 34580 (Thermo Fisher Scientific H21486) in PBS. Following three washes in PBS, coverslips were mounted on microscope slides (VWR^®^ 48312-401) using ProLong™ Gold Antifade Mountant (Thermo Fisher Scientific P36930).

### 4.7. Fluorescent Imaging

Images quantified in Figure 4 were acquired using the Leica DMi8 spinning disc confocal microscope (Leica Microsystems (Leica Microsystems, Wetzlar, Germany)) by a cooled electron-multiplying charged-coupled device (EM-CCD) camera (Hamamatsu (Hamamatsu City, Japan)) with a 40×/1.3 NA oil immersion objective, captured using Volocity^®^ (v6.3) image acquisition software (Quorum Technologies (Quorum Technologies, uslinch, ON, Canada)). Images quantified in Figure 5 and Figure 6 were acquired using the Agilent BioTek Lionhart XL automated microscope (Agilent Technologies (Agilent Technologies, Santa Clara, CA, USA)) at 40X configured by BioTek Gen5 (Agilent Technologies, Winooski, Vermont, USA) (vI+) software.

### 4.8. Fluorescence Intensity Measurements

Measurements of fluorescence intensity of MTs were generated using the selection tools in Fiji (v2.14.0/1.54f). Intensities were captured from single frames or maximum intensity projections, followed by background intensity subtraction from each measurement.

### 4.9. Ciliation Assays

For hTERT-RPE1 cells, 200,000 cells per well were electroporated as previously described and then plated on glass coverslips in 12-well plates. The following day (~90% confluence), the cells were serum-starved in Opti-Mem™ for 48 h to induce ciliation. Immunofluorescence methodology was followed as previously described. The percentage of ciliated cells and the length of primary cilia was visualized using an Agilent BioTek Lionhart XL automated microscope (Agilent Technologies). Multiple ROIs were randomly selected across the coverslip using the DAPI channel. The percentage of ciliated cells was calculated as the number of primary cilia divided by the total number of DAPI-labelled nuclei, as scored manually using Fiji. The length of primary cilia was measured using Fiji using the Measure function on maximum intensity projections of Z-stacks.

## 5. Conclusions

Collectively, the results presented in this study suggest that through CRISPRa genetic modulation, certain β-tubulin isotypes can compensate for variant tubulin protein to overcome certain disease-associated phenotypes. These observations contribute to our knowledge of β-tubulin protein function, tubulin dynamics, and the molecular mechanisms underlying β-tubulin compensation. This study provides a foundational step towards the translation of variant-agnostic genetic therapy for tubulinopathies or patients with primary ciliopathy-like phenotypes with underlying missense variants in TUBB.

## Data Availability

The original contributions presented in this study are included in the article. Further inquiries can be directed to the corresponding author.

## Abbreviations

The following abbreviations are used in this manuscript:

CRISPR	Clustered Regulatory Interspaced Short Palindromic Repeats
sgRNA	Single Guide RNA
TUBB	Tubulin Beta Class I
TUBB1	Tubulin Beta Class VI
TUBB2A	Tubulin Beta 2A Class IIA
TUBB4A	Tubulin Beta 4A Class Iva
TUBB6	Tubulin Beta 6 Class V
dCas9	Dead Cas9
CRISPRa	CRISPR activation
SAM	Synergistic activation mediator
HSF1	Heat shock factor 1
scRNA	Scaffold RNA
hTERT-RPE1	hTERT immortalized retinal pigment epithelial cells
qRT-PCR	Quantitative reverse transcription polymerase chain reaction
ROI	Region of interest
SD	Standard deviation
